# Functionality mediated colour shifting gels from cetyltrimethylammonium bromide and 6-amino caproic acid

**DOI:** 10.1098/rsos.241398

**Published:** 2025-03-05

**Authors:** Chapireddy Nagarjuna, Illa Ramakanth

**Affiliations:** ^1^ Department of Chemistry, School of Advanced Sciences, VIT-AP University, Amaravati, Andhra Pradesh 522241, India

**Keywords:** supramolecular gels, H-bonding, surfactants, charge transfer, lamellar structure, organogels

## Abstract

A charge transfer gel from a cetyltrimethylammonium bromide (CTAB) and an additive 6-aminocaproic acid (6-ACA), in a two-component solvent mixture at a critical solvent composition of 3 : 1 v/v water : toluene is reported. The pale yellow colour of the gel arising from charge transfer interactions between the amine group (–NH_2_) of hexylamine and the ammonium ion (N^+^) of CTAB was confirmed after performing a set of trial experiments with CTAB-heptanoic acid and CTAB-hexylamine systems. The process of gelation, gel phase development and its microstructure were investigated using spectroscopy, microscopy, and small-angle X-ray scattering (SAXS) techniques. The gel formed a lamellar organization while preserving a loose-packed arrangement of a bilayer of CTAB and 6-ACA molecules, maintaining H-bonding along with significant charge transfer interactions. The SAXS pattern revealed a distinct crystalline form of the lamellar gel, indicating a stable phase characterized by alternating layers of crystalline and amorphous structures. This unique gelation nature was inferred from the interplanar spacings, demonstrating its non-conducive properties in highly polar solvents such as methanol and ethanol, as well as in less polar solvents like cyclohexane and carbon tetrachloride, when the organic solvent polarity was altered in the presence of water in the mixed amphiphilic system.

## 1. Introduction

Depending on the surfactant’s molecular organization, type of solvents, additives and its molar ratio, surfactant molecules self-assemble to form organized aggregates in solution. When ionic or co-surfactant additives are present in the surfactant solution, structural transitions are evolved due to the decreased repulsions from the micellar head groups. This causes ionic surfactants to form spherical micelles when their concentration exceeds the critical micelle concentration. Anisotropic in-phase transitions in micellar aggregates provide a variety of aggregate geometries under the conducive circumstances [1]. The practical utility of surfactants requires the appropriate confection of their various mixtures due to the exponential rise of their industrial and technical exploitation. In order to fulfil the needs of commercial applications, actual systems need surfactant mixtures with optimum qualities [2]. One excellent illustration of how bottom-up manufacturing may be used in the construction of nanoscale structures is the usage of supramolecular gel-phase materials that rely on low molecular weight gelators. Supramolecular gels are made up of individual molecules arranged in a self-complementary network of contacts, which allows the molecules to come together into long fibrillary networks and create a self-supporting gel [3]. Molecular self-assembly has been demonstrated to be solvent-dependent and driven by many dynamic non-covalent interactions for a variety of supramolecular structures [4,5].

The formation of a number of two-component gel systems using 4-(4-alkoxybenzoyloxy) 4-stilbazole (nSZ) derivatives and l-tartaric acid has demonstrated the versatility of alkyl chain-substituted stilbazoles for the synthesis of molecules such as pyroelectric Langmuir–Blodgett fabrications, optically nonlinear systems and metallomesogens. However, on their own, these materials are typically unable to self-assemble into supramolecular gels. l-Tartaric acid is a well-known proton donor that interacts with stilbazoles via a number of H-bonds in order to facilitate self-assembly [6,7]. It is widely accepted that through non-covalent interactions such as H-bonding, van der Waals force, π–π stacking and coordination interaction, as well as charge transfer, organogelators having functional groups such as hydroxyl, amide, linear alkyl chains and aromatic groups can self-assemble into aggregates in a variety of morphologies, including fibres, sheets and ribbons [1,8–11]. Due to surface tension and capillary forces, self-assembled ‘one-dimensional’ fibres in a large number of one- and two-component low molecular weight gels may immobilize significant amounts of organic solvents in three-dimensional networks [12,13]. Many organic compounds either crystallize (greater than their saturation concentration) or continue to dissolve (diluted solution) when dispersed in a solvent and allowed to cool. Certain substances are referred to as low molecular weight gelators because they produce supramolecular gels, specifically hydrogelators for aqueous solutions and organogelators for organic solvents as well as oils [14–18]. Multicomponent gel systems are capable of introducing functional groups and so the ease of formation of organogels containing specified functionalities without the need for complex chemical synthesis [19]. Organic solvents cannot usually be gelled in the presence of water in an organogelator as water competes with the H-bonding surfaces of the gelator. The ecology and the ecological balance are seriously harmed by oil spills and hazardous industrial waste derived from solvents [20–25].

When a hot solution of low molecular weight organic compound is cooled below the critical temperature, a gel having viscoelastic material is formed. Low molecular mass organic gelators (LMOGs) are the organic compounds that exhibit this intriguing property of immobilizing solvent molecules, leading to gel formation [26,27]. The LMWG’s self-assembly, which is fuelled by non-covalent interactions such as H-bonding, π–π interactions, metal-ligand coordination, van der Waals forces, hydrophobic effects, etc., is essential to the formation of gels [28–30]. Two-component gels that are metastable in nature gradually transform into a microcrystalline form, perfect for examining molecular aggregation processes and the gel–crystal interface. Since gels are often created by slowly chilling a solution phase, observational studies of phase transitions were critical in understanding the relationship between gelation and crystallization [31–35]. A straightforward and very efficient organogelation method consists of two parts (such as G2-Lys and C6R/S) in a 1 : 1 ratio: (i) a primary amine and (ii) a chiral secondary dendron based on l-lysine (G2-Lys) with a carboxylic acid at the focus point [36]. The ability to move solvents inside the network’s interstices is impeded. Therefore, it is not unusual for there to be a tenfold increase in viscosity caused by only one gelator molecule for every 10^5^ solvent molecules [37]. Organogels are viscoelastic materials that do not flow when a tiny quantity of organogelator (often less than 1 wt%) is added to a large amount of organic solvent [38]. The ability of molecules to interact with each other and create an arrangement that is capable of hierarchically assembling into a gel by simply combining them has made multi-component gels of particular interest. A complex may be formed by non-covalent interactions among the molecular building blocks, or an entirely novel molecule can be created via covalent interactions [39]. In recent days, microplastics, an environmental hazard, are tiny plastic debris that pose a greater threat to the ecosystem and are found to enter into the human bodies through the water and thus endanger aquatic and terrestrial life forms. In order to combat these emerging pollutants, the researchers are interested in designing and fabricating various gels in two-component solvent mixtures utilizing a non-covalent approach to make a strategy of dissolving non-polar microplastics in organic solvents and the polar microplastics in aqueous solvents, forming a smart gel [40].

Cetyltrimethylammonium bromide (CTAB) is prominent for its antiseptic and antibacterial characteristics, whereas 6-aminocaproic acid (6-ACA) has vigorous antifibrinolytic activity [41] and bola-amphiphilic characteristics. Facilitated by ion–dipole interactions, the synthetic combination of a two-component CTAB and 6-ACA leads to very stable molecular gels, followed by an investigation of structural characteristics, which is the first report of its kind. The gelation behaviour of CTAB:6-ACA could be explained by the combined contributions of various intermolecular interactions influenced by gelator concentration and solvent composition. Changes in the composition of the mixed surfactant system may provide novel structures instead of requiring the synthesis of new materials, which makes it technically significant for adjusting microdomain characteristics with minor compositional changes. The multi-component surfactants created gel microstructures that were thermodynamically more stable than those of single-component surfactants due to the significant π-stacking, hydrophobic interactions and dominating charge transfer. Following our recent report on solvent selective gelation of CTAB [42], the objective of the current work is to explore the functionality responsible for charge transfer and the coloured gel formation from CTAB and 6-amino caproic acid. Tuning the functionality in the gelator molecules leads to coloured gels. Understanding the chemistry of coloured gels enables us to design the gelator molecules with a specific functionality and thus inspires an approach for creating gels with natural flavours instead of using artificial ones.

## 2. Material and methods

**Scheme 1.**
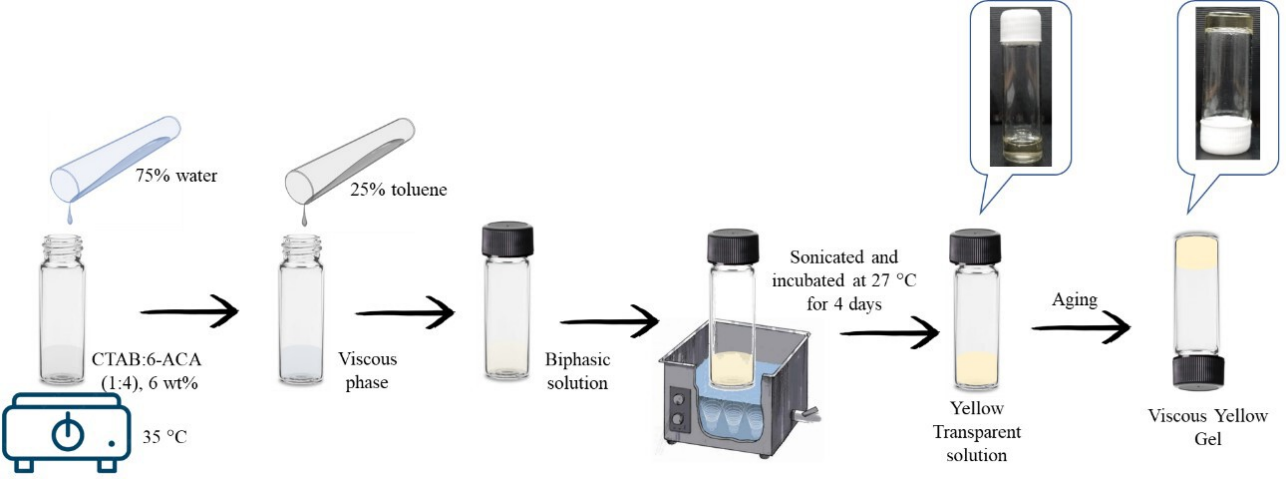
Methodology of CTAB:6-ACA gel formation in a 75 : 25 v/v water : toluene solvent mixture.

We have purchased 99% pure CTAB from SRL Pvt. Ltd. in India. We also received 99.5% pure toluene and 99% pure 6-ACA from the same source. A digital conductivity meter with a cell constant of 1 cm^−1^ (Mettler Toledo FiveEasy Plus FP30, Switzerland) was used to test conductivity. For making the aqueous solutions, ultrapure deionized water (LAB-Q, India) kept at 25 ± 0.1°C was used. A Bexco UV quartz cuvette with a 3.5 ml path size was used with a Shimadzu UV 1900 double-beam spectrophotometer (Japan) to record electronic spectra. Scheme 1 has specifics about the gel formation process.

**Small-angle X-ray scattering (SAXS)** investigations of gel samples were done employing Bruker-AXS NanoSTAR equipment (Germany). The X-ray source exploited CuKα emission at an estimated 45 kV/100 mA. The SAXS research spans the *q* range of 0.05–0.72 Å⁻¹, comparable to a 2*θ* range of 0.05 and 12.5° [42].

**X-ray diffraction** measurements were conducted utilizing Empyrean equipment (UK) using CuKα radiation. The measurements included a 2*θ* range from 5° to 120° [42].

**Scanning electron microscopy (SEM)** images were taken employing Zeiss equipment (Germany). The samples were coated with carbon utilizing a vacuum sputter coater and then desiccated at 25°C under decreasing vacuum circumstances. Imaging was achieved by employing an electron beam that varied from 0.02 to 20 kV to analyse the materials [42].

**Optical microscopy** pictures were taken using a Nikon microscope (Japan) fitted with CFI TU PLAN FLOUR lenses for brightfield and darkfield viewing, precisely the Eclipse LV150N type [42].

**Transmission electron microscopy** pictures were taken using a Philips CM12 instrument. Sample preparation was done on a carbon-coated Cu substrate, which permitted spontaneous evaporation at ambient temperature [1].

**Rheology** The gel formation multi-phase characteristics have been investigated using an Anton Paar 100 Rheometer (Austria). The rheometer had been fitted with a plane geometry at a cone angle of 2°, working at 25°C with a vibratory frequency of 1 Hz as well as a pressure magnitude of 50 Pa [1].

## 3. Results and discussion

CTAB is a cationic surfactant that forms spherical micelles in water at a CMC of 0.97 mM [42], but preferentially forms reverse micelles in toluene. When a heteroditopic bola-amphiphile, such as 6-ACA, was introduced to the reverse micellar solution of CTAB in a 1 : 4 stoichiometric proportion throughout a predetermined composition range, vesicular and gelation phases were seen in the two-component solvent mixture. Considering the building blocks of amino acids, which comprise biological systems, the carboxyl-terminated amines were observed to be the preferred option. The ratio of stoichiometry among CTAB and its bola-amphiphile controlled the shape of the self-assembly. In addition to an aqueous 6-ACA solution to a CTAB reverse micelle in toluene and sonication, the reverse micelles transformed from a low viscous solution to a strong gel appearing yellow in colour with ageing. Bile salt and sodium deoxycholate added to sodium bis(2–ethylhexyl) sulfosuccinate (AOT) reverse micellar solutions were reported to induce a phase shift between low-viscosity liquids and stretchable and stiff organogels [43].

**Figure 1.**
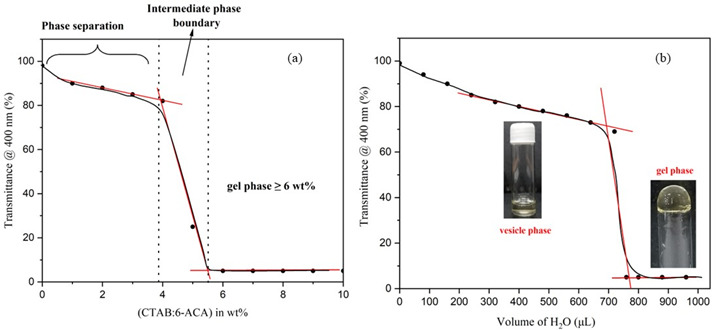
(a) UV-visible measurements in transmittance mode as a function of CTAB:6-ACA concentration indicating the formation of CTAB:6-ACA gels. (b) UV-visible transmittance indicating intermediate vesicle phase with the formation of a gel phase upon the addition of water required for gel formation. Insets show the CTAB:6-ACA gel’s stable-to-inversion method along with the vesicle phase in the solvent mixture of water : toluene.

The CTAB:6-ACA gel phase is identified by the inversion of a vial method and confirmed by the UV-visible transmittance measurements. Figure 1a indicates the essential concentration required for a CTAB:6-ACA gel formation, which is observed to be approximately 6 wt% in a two-component solvent mixture of water : toluene, as observed from UV-visible transmittance measurements. Based on the transmittance measurements with different water contents, the required quantity of water for gel formation was found to be 750 *µ*l, as shown in figure 1b. In order to conduct these studies, a 1 : 4 mole ratio of CTAB:6-ACA was heated to 40°C in toluene. In each experiment, the above combination was mixed with ultrapure water (a few microlitres), sonicated for a few minutes and then slowly cooled. The sample vials were tightly closed to prevent toluene from evaporating. CTAB:6-ACA formed a clear, non-viscous solution in the dark after 1 week of incubation at 27°C. Further, it transformed into a viscous, stable, pale yellow-coloured gel. The as-formed gel was observed to be optically transparent. Spherical unilamellar vesicles were formed prior to the gel phase formation at less than 6 wt% of 1 : 4 CTAB:6-ACA viscous solution.

**Figure 2.**
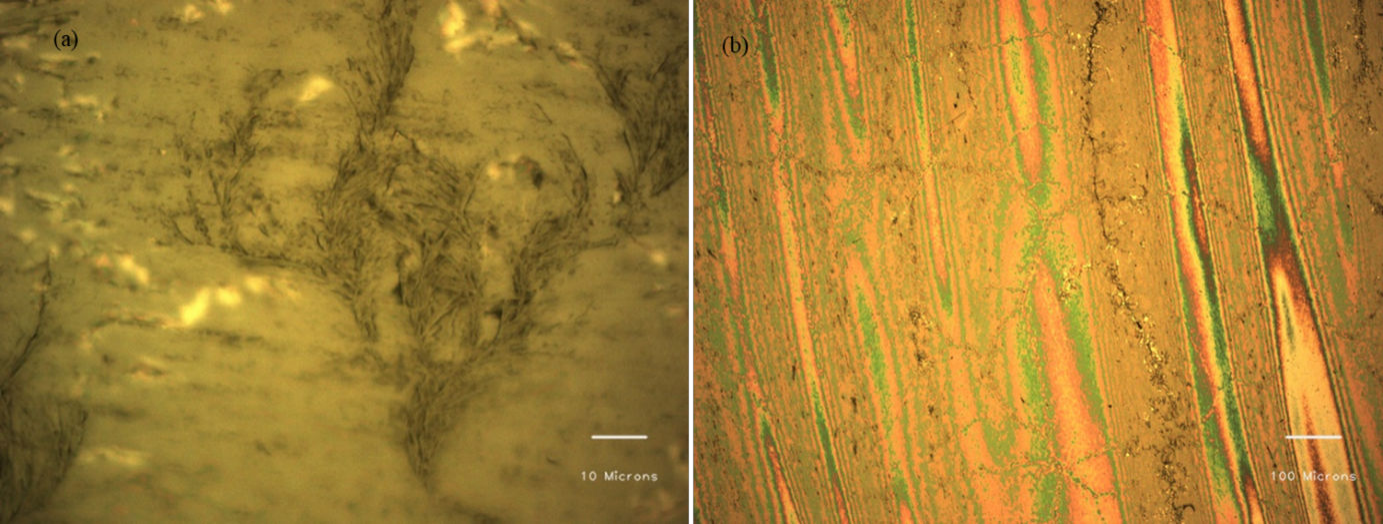
(a) CTAB:6-ACA gel phase under polarizer mode (10×). (b) Optical image of the xerogel exhibiting birefringence under a polarizer (100× magnification).

**Figure 3.**
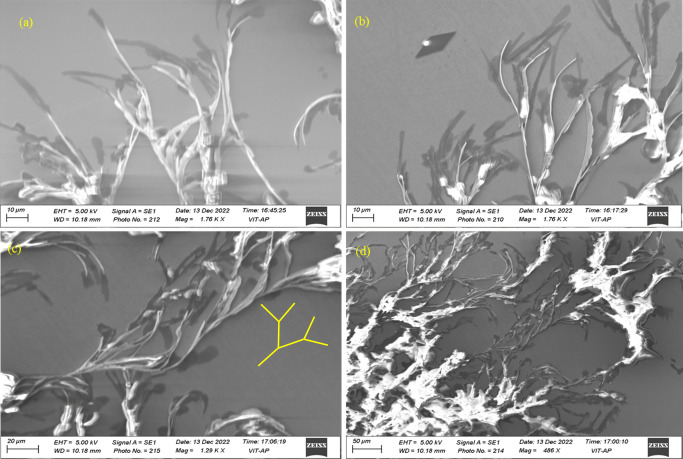
SEM images of CTAB:6-ACA gel showing fibrillar networks similar to Cayley treelike structures with 10–100 *µ*m width [44].

**Figure 4.**
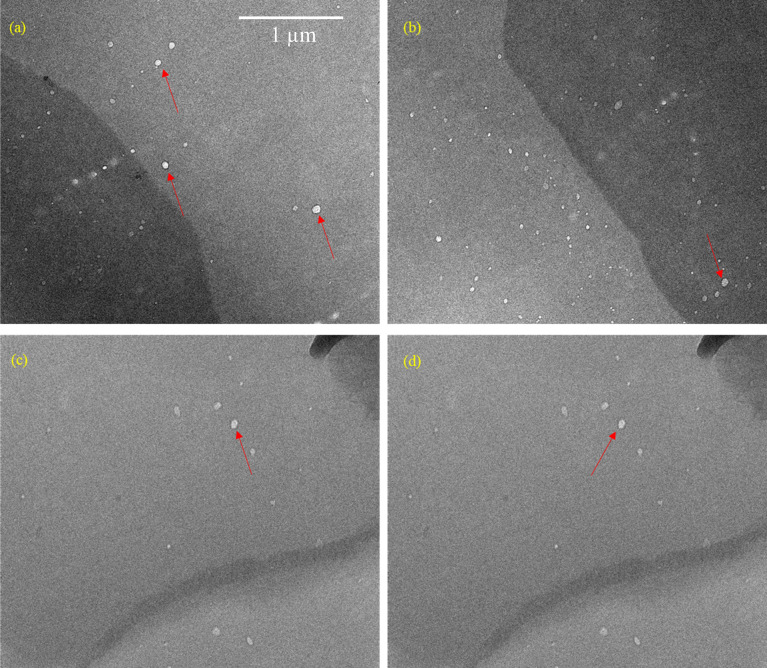
TEM images of unilamellar vesicles of size 5–70 nm formed from CTAB:6-ACA.

Turbidity measurements of the CTAB:6-ACA toluene solution with gradual addition of water were performed. Figure 1b reveals an abrupt decline in transmittance, indicating the development of a gel phase. The ‘stable-to-inversion of a vial’ technique was used to examine CTAB:6-ACA gelation ability for different organic solvents with water. The polarizing microscopic images revealed that the xerogels had considerable birefringence, as seen from figure 2b. Several entangled fibres with high aspect ratios, widths of 10–100 *µ*m, and lengths of several micrometres formed extended fibrillar networks similar to Cayley treelike structures [44] evidenced from the SEM images of the CTAB:6-ACA gel, as shown in figure 3a–d. Figure 4a–d shows TEM images of CTAB:6-ACA unilamellar vesicles of 5–70 nm in size.

**Figure 5.**
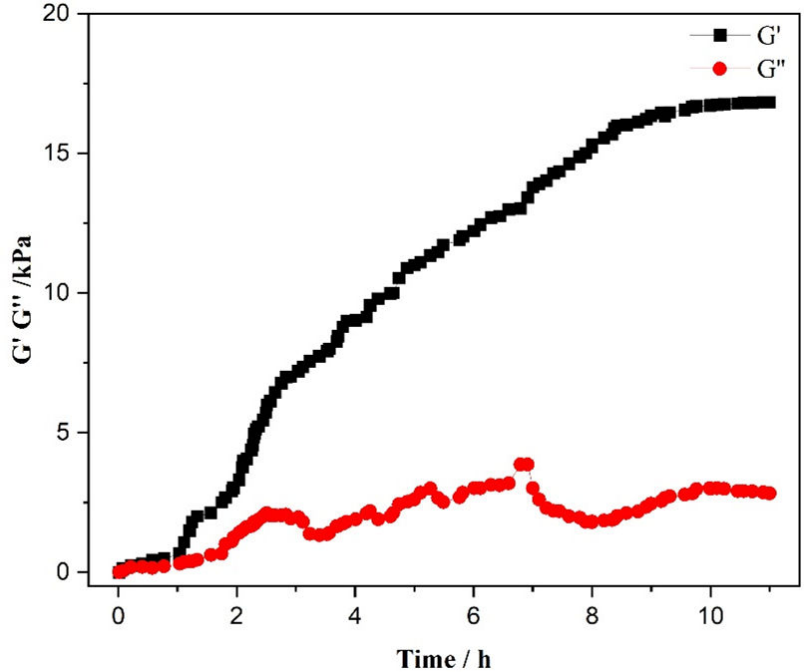
Rheology profiles indicating the variations in *G*′ and *G*″ over time during gel formation in a CTAB:6 ACA gel in a water : toluene combination (3 : 1 v/v).

Rheological investigations were carried out to study the viscoelastic features of gels consisting of CTAB:6-ACA in a two-component solvent combination of water : toluene. Those molecular gels display viscoelastic behaviour, defined by the storage modulus (*G*′) as well as the loss modulus (*G*″), which indicate energy storage and dissipation accordingly. Figure 5 shows the rheological data observed for CTAB:6-ACA in water : toluene (3 : 1 v/v). Initial *G*′ and *G*″ measurements revealed a behaviour analogous to a viscous liquid. With more extended gelation periods (duration > 1 h), there was a considerable rise in *G*′, greatly exceeding *G*″, suggesting the creation of a fibrous oriented bundle structure [45]. Subsequently, *G*′ and *G*″ reached a plateau phase after a few hours, with both moduli progressively rising over time and displaying independent behaviour from 9.5 h onwards. For the CTAB:6-ACA system, cole–cole plots are not obtained, which are indicative of the absence of Maxwellian behaviour [46] of the CTAB:6-ACA solutions. Hence, a reverse micellar phase exists instead of a worm-like micellar phase in CTAB:6-ACA solutions. Further, the system undergoes a transition from reverse micelles to vesicles, then to elongated vesicles, and finally to a fibrous network gel phase that is evident upon the addition of water.

At room temperature, the gel retains stability without affecting its appearance for at least 12 months. Gelation did not occur in very polar solvents like methanol and ethanol, nor in severely non-polar solvents such as cyclohexane and carbon tetrachloride. Table 1 describes how solvent polarity affects gel formation.

**Table 1.** The impact of solvent variation on the formation of CTAB:6-ACA gel under conditions with 75% v/v water.

the dielectric constant of the solvent	appearance
ethanol (*ε* = 24.5)	clear solution
tetrahydrofuran (*ε* = 7.6)	clear solution
methanol (*ε* = 32.7)	clear solution
acetonitrile (*ε* = 37.5)	clear solution
carbon tetrachloride (*ε* = 2.2)	phase separation
cyclohexane (*ε* = 2.0)	phase separation
chloroform (*ε* = 4.8)	weak gel
dichloromethane (*ε* = 9.0)	weak gel
toluene (*ε* = 2.4)	transparent gel

**Figure 6.**
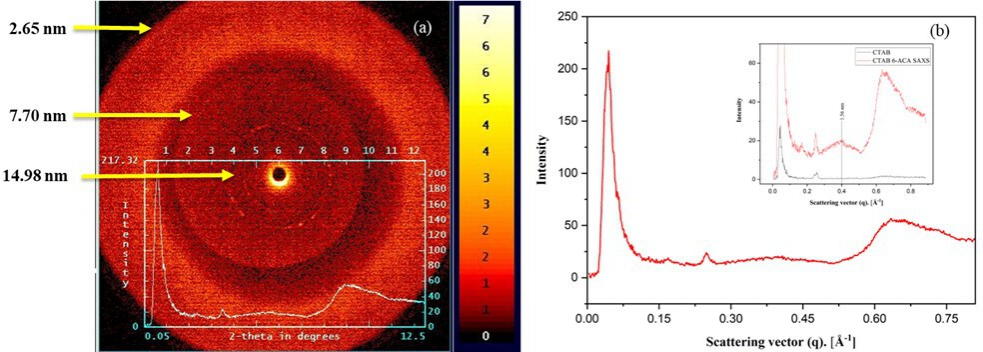
(a) SAXS pattern and (b) spectrum of CTAB:6-ACA gel formation at 25°C. The image indicates the d-spacings derived from *d* = *λ*/2 sin *θ*. The inset shows the comparison of CTAB and CTAB:6-ACA.

SAXS was used to study the internal structure of a CTAB:6-ACA gelation in water : toluene (3 : 1 v/v). A hexagonal (1, 1/√2, 1/√3) packing [47] and a lamellar (1, 1/2, 1/3) packing were differentiated using the distance between the subsequent SAXS peaks. The computed interplanar spacings from periodic diffraction peaks and SAXS profiles in figure 6 are observed to maintain a ratio of 1 : 1/2 : 1/3, suggesting that the gel assembles into an organized lamellar structure [48]. The interplanar distances computed from the scattering vectors, as well as low-angle scattering intensities, are shown in tables 2 and 3, respectively. Weiss and colleagues described a similar experiment for organogels made from primary alkyl amines for latent gelators containing neutral triatomic molecules [49]. The molecular lengths and ‘*d*’ values were compared to analyse how molecules pack within the gel network.

**Table 2.** Interplanar spacings (*d*) calculated from the small-angle peaks in SAXS of CTAB:6 ACA gels.

scattering angle (2 θ/ °)	interplanar spacings, *d* = λ /(2 sin θ ) (nm)
0.57	15.56
0.63	14.13
0.68	13.05
0.89	9.93
1.15	7.70
1.30	6.81
3.50	2.65

**Table 3.** Interplanar spacings (*d*) calculated from the scattering vectors in SAXS of CTAB:6 ACA gels.

scattering vector (*q*), A˚^−1^	intensity (*I*)	interplanar spacings, *d* = **2** π /*q* (nm)
0.0419	203.02	14.98
0.0440	217.32	14.27
0.0490	178.42	12.81
0.0645	79.48	9.81
0.0810	40.77	7.70
0.0953	29.90	6.58
0.2481	24.29	2.65
0.4024	19.09	1.56
0.6387	54.45	0.98

### 3.1. Modelling of the CTAB:6-ACA gelation

**Scheme 2.**
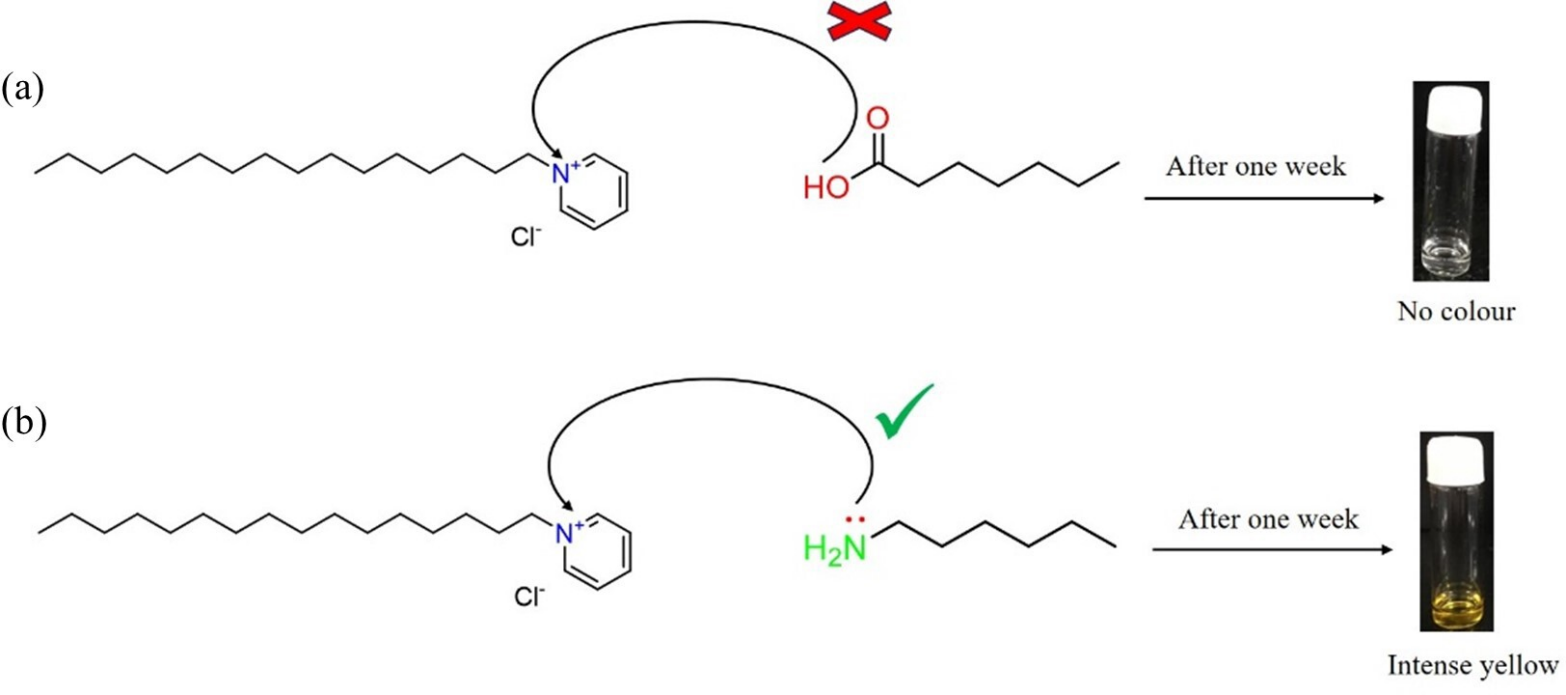
(a) Absence of charge transfer from heptanoic acid to CPC indicating a weak electron donor and (b) presence of charge transfer from hexyl amine to CPC indicating a strong electron donor.

**Figure 7.**
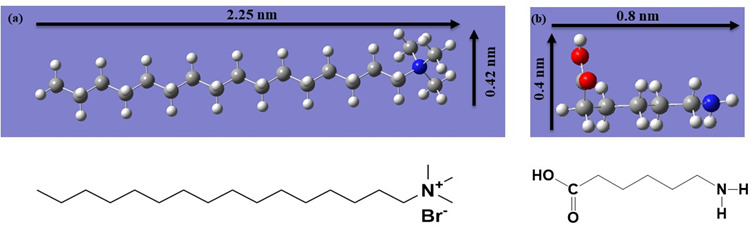
(a) CTAB and (b) 6-ACA, obtained through density functional theory (DFT) calculations using the B3LYP/6-31G++method. The chemical structure corresponding to each molecular model is also depicted.

**Figure 8.**
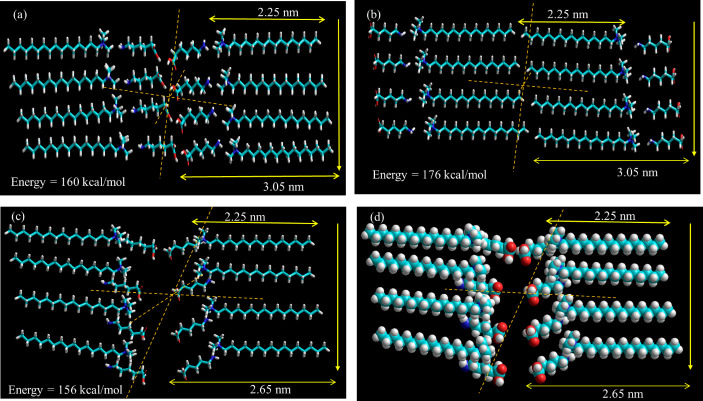
The figure depicts (a) head-to-head, (b) tail-to-tail, (c) loose-packed arrangement and (d) the CPK representation of loose-packed CTAB bilayer assemblies. Each figure shows two pairs of CTAB:6-ACA surfactant units.

Energy-minimized molecular models of CTAB and 6-ACA are depicted in figure 7a,b. SAXS investigations revealed three probable molecular packings that developed a bilayer assembly with periods 2.65 and 3.05 nm, viz. (a) head-to-head, (b) tail-to-tail and (c) loose-packed arrangement is shown in figure 8. The solvent gets exposed by the hydrophilic ammonium head groups of CTAB in the first model shown in figure 8a. In the second one, the bilayer membrane’s outside and inside regions are occupied, accordingly, by the hydrophobic long alkyl chains and hydrophilic ammonium head groups (figure 8b). Both the bilayer membrane’s inner and outer layers are occupied by the hydrophobic long alkyl chains and the hydrophilic ammonium head groups, respectively, in the third configuration as shown in figure 8c. Figure 8 depicts the bilayer structures that were created by optimizing each of the CTAB and 6-ACA molecules using MM+force field. For molecular modelling, an all-trans configuration’s alkyl chain spacing was maintained at 0.45 nm [50]. A loose-packed arrangement molecular structure with a minimal overall energy of 156 kcal mol^−1^ (figure 8c) was observed. Therefore, the lamellar are bilayer structures made up of 6-ACA and CTAB bilayers, where hydrophobic interactions partly interdigitate the molecules. Conversely, the stabilizing contacts mainly consist of (i) charge transfer interactions among the lone pair of electrons on the amino group end of 6-ACA and the ammonium-containing N^+^ of the CTAB molecule; (ii) H-bond interactions between the carboxylic (–COOH) head groups and (iii) hydrophobic interactions. Scheme 2 depicts the influence of weak and strong electron-donating groups on the cetylpyridinium chloride (CPC) acceptor molecule [1].

**Scheme 3.**
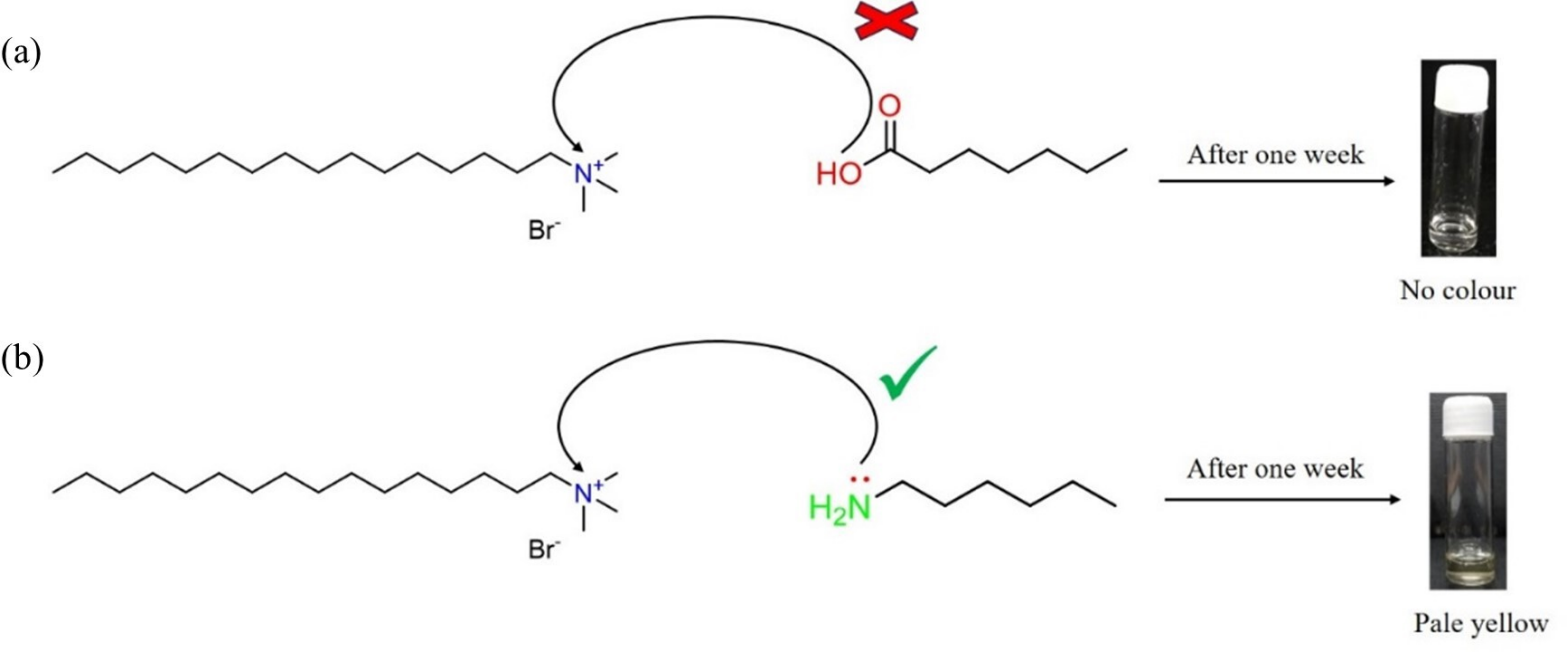
(a) Absence of charge transfer from heptanoic acid to CTAB indicating a weak electron donor and (b) presence of charge transfer from hexyl amine to CTAB indicating a strong electron donor.

In order to confirm whether the pale yellow colour formation is due to the charge transfer from the amine (–NH_2_) group or the carboxylic acid (–COOH) group, we have performed two sets of trial experiments, one with the CTAB-heptanoic acid system and the other with the CTAB-hexylamine system, respectively, with the same two-component solvent mixtures of water : toluene maintaining the same ratio as 3 : 1. In the first set of experiments, an aqueous solution of CTAB was mixed with heptanoic acid, and the mixed solution was allowed to stay for more than a week to stabilize. It was observed that no colour was developed in the gelation phase on ageing, whereas, in the second set of experiments, an aqueous solution of CTAB was mixed with hexylamine, and the resulting solution was allowed to stabilize for more than a week. A pale yellow colour was developed in the case of the CTAB-hexylamine system, indicating that the colour change observed is due to charge transfer from the amine group (–NH_2_) of hexylamine to the ammonium ion (N^+^) of CTAB. Scheme 3 depicts the influence of weak and strong electron-donating groups on the CTAB acceptor molecule.

**Figure 9.**
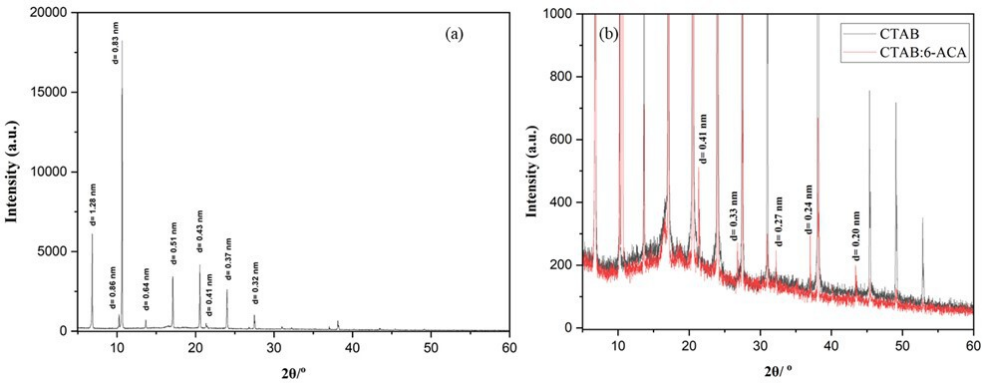
(a) XRD profile of the gelation from CTAB:6-ACA. (b) Comparison of XRD profiles of CTAB and CTAB:6-ACA gels.

Furthermore, the lengthy alkyl chains provide a highly structured layer by a prolonged interdigitated hydrophobic contact, as shown by the (CPK) model structure in figure 8d. The wide-angle area in figure 9 XRD diagram for the CTAB:6-ACA gels, which displays a number of distinct reflection peaks over an ambiguous background, is indicative of this. These peaks accentuate the crystalline structure of the gel. Hence, the formation of CTAB:6-ACA gels as a three-dimensional network with a loose-packed arrangement sustained by charge transfer, H-bond and hydrophobic interactions was validated by the SAXS results. The findings are consistent with the molecular packing of the aggregate structure seen in sugar-based gelators, as described by Jung *et al*. [51]. Estroff *et al*. described the process by which bis-urea dicarboxylic acid-based compounds produce organic hydrogels [52].

### 3.2. Mechanism of gel formation

**Figure 10.**
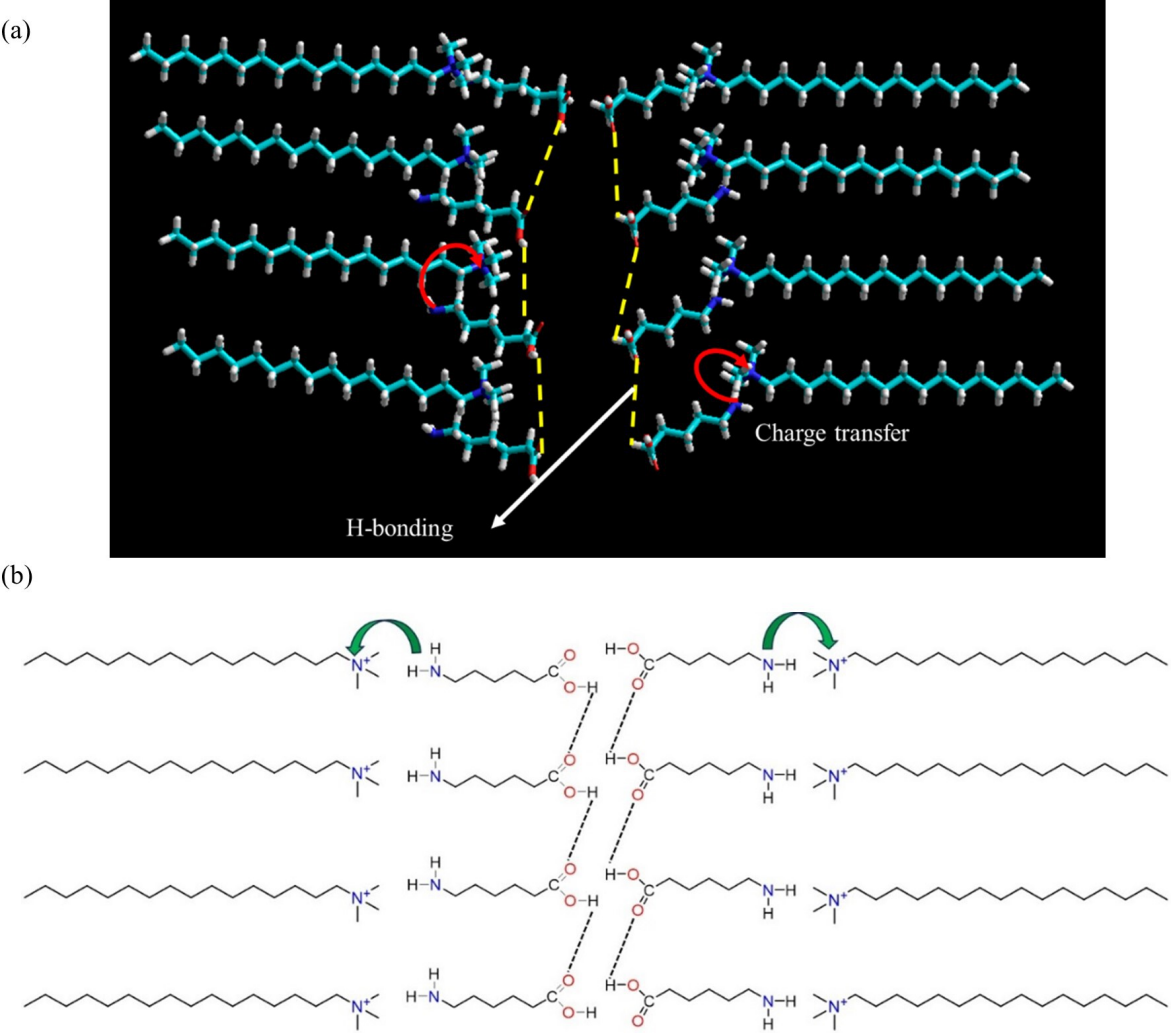
A CTAB:6-ACA (a) molecular model and (b) mechanism reflecting the charge transfer between the amino group of 6-ACA and the quaternary N^+^ that forms the cetyltrimethylammonium ion, as well as the H-bonding between the carbonyl (C=O) and OH groups of the carboxylic acid group. The figure represents primary (1°) and secondary (2°) structures observed in a CTAB:6-ACA (1 : 4) gel.

Figure 10 depicts the extended polymeric network of CTAB:6-ACA that was unravelled from molecular modelling within the gel fibre. One may imagine primary, secondary and tertiary structures resembling proteins on a gel. In the present study, long fibres composed of 6-ACA and CTAB bilayers are reported. The secondary structures have been formed by the fibres subsequent to H-bonding with water molecules. The molecular arrangement of gel affects its secondary structure, which might take the form of micelles, vesicles, ribbons, sheets, etc [53]. The post-aggregation of structures generated from secondary structures and the tertiary structure that makes up the gel is essential in determining whether a molecule may form a gel or precipitate into the solvent [54]. It was reported that charge transfer occurred in a dual-component sugar-based organogel that had donor groups and an acceptor that carried saccharides [55].

**Figure 11.**
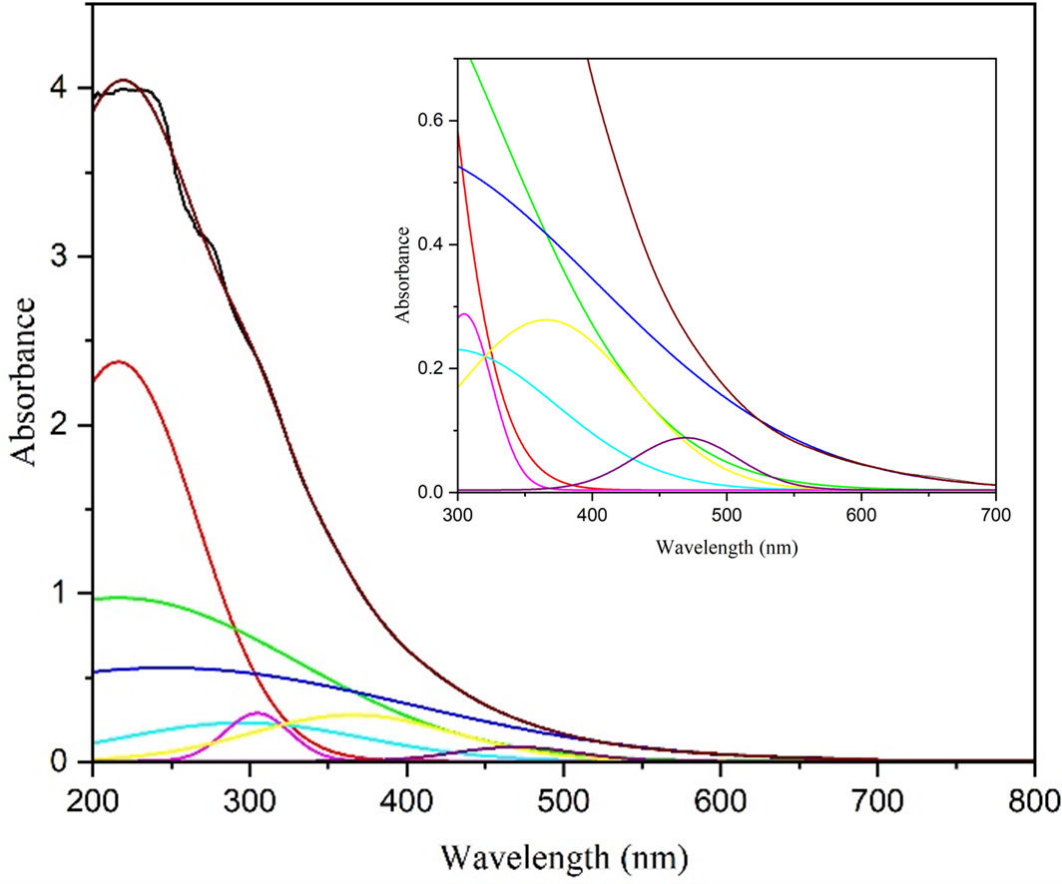
The UV-visible absorption spectrum characteristics of a 1 : 4 CTAB:6-ACA gel in water : toluene two-component solvent mixture, with experimental (black) and Gaussian fitted (coloured) curves. The extended spectrum featuring the distinctive charge transfer band at 483 nm is shown in the inset.

In the current investigation, fibrillar networks formed from CTAB and 6-ACA may form a bilayer. These fibrillar networks form secondary structures when they continue to form H-bonds with water molecules. The charge transfer nature of the gelation at 483 nm is confirmed by the observed UV-vis spectrum, which appears in figure 11.

## 4. Conclusions

We report herein a pale yellow-coloured gel from a 3 : 1 v/v water : toluene critical two-component solvent composition at 6 wt% of 1 : 4 CTAB:6-ACA. The self-assembling characteristics of CTAB:6-ACA were investigated for various solvent combinations. Addition followed by ultrasonication of toluene solution of CTAB to an aqueous solution of 6-ACA resulted in a structural change from reverse micelles to vesicles to gels. It is observed that the addition of 6-ACA to CTAB resulted in a colourless to a pale yellow-coloured gel and further stabilized the gel. The structural and the dynamical characteristics of the CTAB:6-ACA gel system were investigated using molecular modelling, UV-visible measurements in transmittance mode, optical, polarizing microscopy, SAXS and SEM. A predominant charge transfer and H-bonding interactions were observed through several trial experiments separately using heptanoic acid, hexyl amine and cetylpyridinium chloride molecules. Analysing SAXS spectra coupled with molecular modelling showed that the gels formed a three-dimensional network that stabilized the loose-packed arrangement bilayer of CTAB and 6-ACA molecules via a thickness of 2.65 nm. Hydrophobic and H-bonding interactions also supported the formed structure. The gel showed a lamellar structure, confirmed by periodic diffraction peaks observed in SAXS analysis. These molecular gels offer tailored microenvironments conducive to controlled electrochemistry of redox-active molecules. Modulating the functional groups in precursor molecules results in the formation of a diverse range of coloured gels. The structure of the as-formed gel, viz crystalline, semi-crystalline and amorphous gel, depends on choosing the right groups (aliphatic/aromatic) in the precursor molecules. These smart gels could be utilized for trapping polar and non-polar microplastics. These colour-shifting gels provide a platform for creating materials that respond dynamically to environmental stimuli, such as pH, ionic strength or temperature. This responsiveness can be tailored for sensors, smart coatings and advanced display technologies.

## Data Availability

Our data deposited at Dryad [56]. Supplementary material is available online [57].
